# Thymoquinone exerts potent growth-suppressive activity on leukemia through DNA hypermethylation reversal in leukemia cells

**DOI:** 10.18632/oncotarget.16431

**Published:** 2017-03-21

**Authors:** Jiuxia Pang, Na Shen, Fei Yan, Na Zhao, Liping Dou, Lai-Chu Wu, Christopher L. Seiler, Li Yu, Ke Yang, Veronika Bachanova, Eric Weaver, Natalia Y. Tretyakova, Shujun Liu

**Affiliations:** ^1^ The Hormel Institute, University of Minnesota, Austin, MN 55912, USA; ^2^ Department of Hematology, Chinese PLA General Hospital, Medical School of Chinese PLA, Beijing 100853, China; ^3^ Department of Biological Chemistry and Pharmacology, The Ohio State University, Columbus, OH 43021, USA; ^4^ Department of Medicinal Chemistry, University of Minnesota, Minneapolis, MN 55455, USA; ^5^ Chongqing Engineering Research Center of Stem Cell Therapy, The Children's Hospital of Chongqing Medical University, Chongqing 400014, China; ^6^ Division of Hematology, Oncology and Transplantation, Minneapolis, MN 55455, USA; ^7^ Prairie Pharms LLC, Nora Springs, IA 50458, USA

**Keywords:** DNA methylation, leukemia, thymoquinone, DNA methyltransferase, bioactive compounds

## Abstract

Thymoquinone (TQ), a bioactive constituent of the volatile oil of *Monarda fistulosa* and *Nigella sativa*, possesses cancer-specific growth inhibitory effects, but the underlying molecular mechanisms remain largely elusive. We propose that TQ curbs cancer cell growth through dysfunction of DNA methyltransferase 1 (DNMT1). Molecular docking analysis revealed that TQ might interact with the catalytic pocket of DNMT1 and compete with co-factor SAM/SAH for DNMT1 inhibition. *In vitro* inhibitory assays showed that TQ decreases DNMT1 methylation activity in a dose-dependent manner with an apparent IC50 of 30 nM. Further, exposure of leukemia cell lines and patient primary cells to TQ resulted in *DNMT1* downregulation, mechanistically, through dissociation of Sp1/NFkB complex from *DNMT1* promoter. This led to a reduction of DNA methylation, a decrease of colony formation and an increase of cell apoptosis via the activation of caspases. In addition, we developed and validated a sensitive and specific LC-MS/MS method and successfully detected a dynamic change of TQ in mouse plasma after administration of TQ through the tail vein, and determined a tolerable dose of TQ to be 15 mg/kg in mouse. TQ administration into leukemia-bearing mice induced leukemia regression, as indicated by the reversed splenomegaly and the inhibited leukemia cell growth in lungs and livers. Our study for the first time demonstrates that DNMT1-dependent DNA methylation mediates the anticancer actions of TQ, opening a window to develop TQ as a novel DNA hypomethylating agent for leukemia therapy.

## INTRODUCTION

Acute myeloid leukemia (AML) is a highly aggressive hematologic malignancy characterized by an uncontrollable proliferation of immature myeloid blasts. While intensive chemotherapy, usually consisting of an anthracycline and cytarabine, induces remission in about 50% of older patients, most of these patients still succumb to their disease. New approaches improving outcome in AML are highly needed. Overexpression of DNA methyltransferases (DNMTs) is frequent and changes in DNA methylation are a constant feature of AML [1–3]. Consequently, abnormal DNA methylation has been an attractive target for therapy in AML. Inhibitors for DNMT1, such as azacitidine and decitabine, have entered into clinical trials for AML patients [4–6]. An accepted mechanism for the anti-tumor activity of these agents is their incorporation into newly synthesized DNA strands followed by covalent binding, sequestration and depletion of the DNMT1 [6–8]. Although positive clinical outcomes have been achieved in certain patients, the prognosis remains poor due to the relapse and/or non-responsiveness. Mechanistically, such high failure rate of therapy could result from the drawbacks of decitabine, including cell cycle-dependent activity, low efficiency of DNA incorporation [9] and lack of obvious inhibitory effects on DNMT3a and DNMT3b [10]. Thus, the development of novel DNA hypomethylating compounds with different action mechanisms may broaden the therapeutic toolbox targeting epigenetic abnormality in AML.

Natural products from plants are becoming more and more interesting, because they are relatively non-toxic, inexpensive and available in an ingestive form [11] with significant anticancer effects. Preclinical and clinical studies demonstrated that the anticancer properties of bioactive components (i.e., pathenolide, folate, retinoids etc.) may be attributed to its influence on epigenetic processes through binding to DNMT1 enzymatic center or/and disrupting *DNMT* transcription [12]. TQ is one of the most bioactive ingredients of *Nigella Sativa* seeds, which is regarded in the Middle East as part of an overall holistic approach to health and is thus incorporated into diets and everyday lifestyles. TQ is also found in high concentration in the prairie plant *Monarda fistulosa*, known as wild bergamot. It is reported that TQ exerts significant anti-neoplastic activity against human cancers [13], anti-oxidant effects and anti-inflammation in animal models and cell culture systems [14, 15], chemopreventive effects, most importantly, the specific growth inhibitory effects on cancer cells, not normal cells [16, 17], and anti-multidrug-resistant variants of human malignant cell [13]. However, how TQ manifests these activities is not fully understood, although it has been shown to downregulate the expression of *Bcl-xL* [18], *COX-2* [19], *iNOS* [20], *5-LOX* [21], *TNF* [22] and *cyclin D1* [16], all known to be regulated by NFkB activity that can be blocked by TQ [23]. Given the regulatory role of NFkB in *DNMT1* expression [1, 2, 24], these investigations support the notion that TQ may influence epigenetic events in cancer cells, which has not been studied.

In the current report, we investigated the molecular mechanisms of TQ anti-leukemia actions. We demonstrate that TQ binds to the DNMT1 catalytic site leading to inhibition of DNMT1 enzymatic activity, and that TQ mediates *DNMT1* downregulation, at least partially, through disruption of Sp1/*miR-29b* loop resulting in a reduction of DNA methylation. Consequently, TQ promoted cell growth arrest, apoptosis *in vitro* and *ex vivo* and induced leukemia regression in mice. These findings support TQ as an additional DNA methylation modulator that mechanistically differs from conventional hypomethylating agents.

## RESULTS

### Molecular modeling of TQ binding to DNMT1 catalytic site

**Figure 1:**
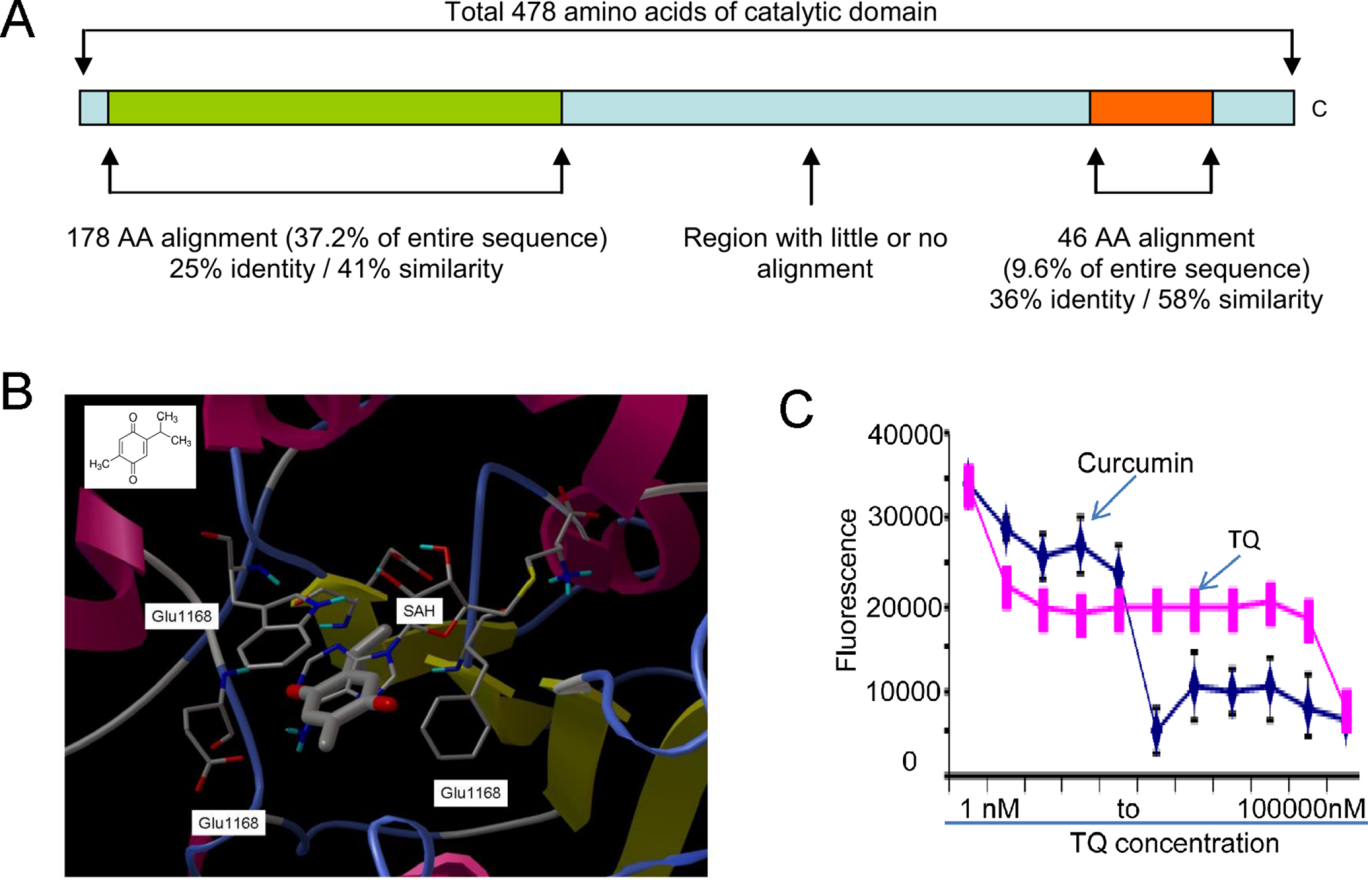
TQ binds to DNMT1 catalytic pocket (**A**) Schematic diagram of the alignment of bacterial modification methylase (Hhal) catalytic domain (PDB ID: 4MHT) against the 478 amino acids (AA 1139-1616) of DNMT1. (**B**) TQ (thick ball-and-stick) binding to DNMT1 catalytic site (ribbon representation). Important binding site residues are shown as thin ball-and-stick and labeled, cofactor SAH shown as thin ball-and-stick and TQ structure shown at upper left corner. TQ largely competes with cofactor SAM/SAH for DNMT1 inhibition. (**C**) *In vitro* inhibitory assay of TQ on the enzymatic activity of M. SssI. Data are mean ± SD; *p < 0.01.

It is well known that the dietary phytochemicals (e.g., curcumin, lycopene, genistein) reverse abnormal DNA methylation landscape in various types of cancer [25, 26], but it is unknown whether TQ, another phytochemical compound, possesses inhibitory activity on DNMT1-dependent DNA methylation. To address this, a homology model of human DNMT1 catalytic domain was initially built with the crystal structure of bacterial modification methylase (Hhal) catalytic domain (PDB ID 4MHT) as the modeling template (Figure 1A). AutoDock version 4.051 was used for the docking simulation. Figure 1B showed simulated bindings of TQ and cofactor SAH onto the catalytic site of DNMT1 homology model. The DNMT1 catalytic site was a deep pocket buttressed by a typical pseudo-Rossmann fold in the bottom and walled by helices and loops. The pocket was largely hydrophobic with polar residues in the binding sub-pockets on the methionine end of the cofactor and at the side of pyrimidine aromatic ring of the substrate. TQ binding competes mainly with the adenosine side of cofactor. The phenyl ring was sandwiched between Trp1136 and Phe1111 via strong aromatic interaction, and one of the carbonyl groups was H-bonded to Glu1168 main-chain amide. These results support the potential of TQ to inhibit DNMT1 catalytic function.

### TQ inhibits DNMT1 activity *in vitro*

As TQ was predicted to bind to the DNMT1 catalytic site, we reasoned that TQ would ablate DNMT1 activity. To test this, we performed *in vitro* inhibitory assay [25] of TQ on the enzymatic activity of M. SssI, an analog of DNMT1 with robust methylation activity. Its catalytic domain was structurally similar to DNMT1. In brief, we used a 38 bp double stranded (ds)-oligonucleotide containing the sequence CCGG, which was labeled with 3′-biotin in one strand and 3′-digoxigenin-NHS ester in its complementary strand. When CCGG was methylated (CC^m^GG), HpaII was unable to cleave it resulting in the generation of fluorescence signal that was positively correlated with M. SssI enzymatic activity. As shown in Figure 1C, exposure to various concentrations (1, 10, 30 and 300 nM, 1, 3, 10, 30 and 100 μM) of TQ led to a dose-dependent decrease in fluorescent intensity, reflecting inhibition of the M. SssI methylation activity. Curcumin, another DNA methylation modulator [25], was used as a positive control. The apparent IC_50_ of TQ with respect to M. SssI inhibition was 30 nM. Although the exact binding mode of TQ to DNMT1 catalytic center needs further exploration, these data support the inhibitory effect of TQ on DNMT1 partially through protein binding.

### TQ abrogates *DNMT1* expression through Sp1/*miR-29b* negative feedback loop

**Figure 2:**
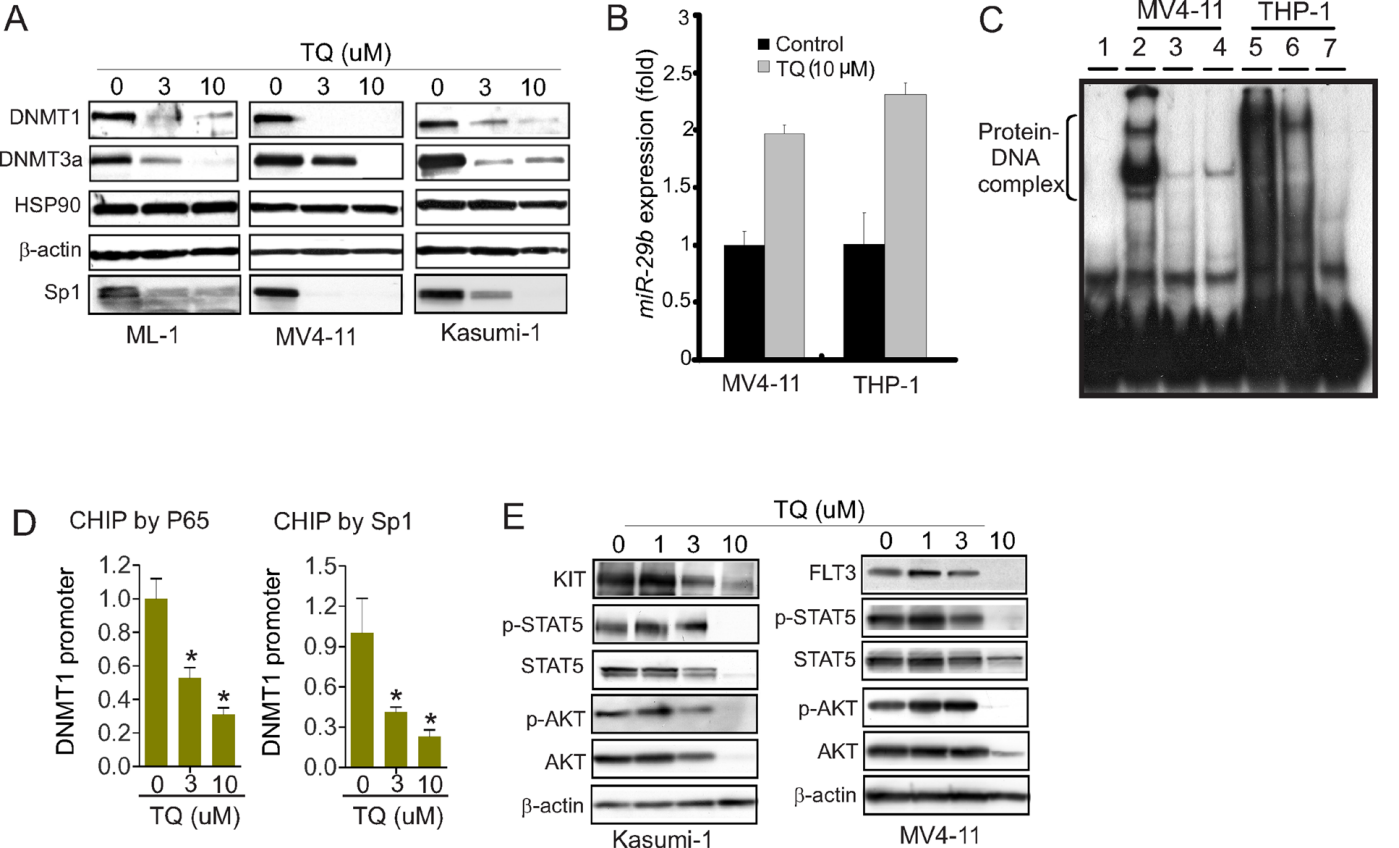
Treatment with TQ suppresses DNMT1 expression (**A**) Western blot in ML-1, MV4-11 and Kasumi-1 cells treated with indicated amounts of TQ for 24 hours. (**B**) qPCR measuring *miR-29b* expression in MV4-11 and THP-1 cells upon exposure to TQ for 3 hours. (**C**) Nuclear extract from MV4-11 and THP-1 cells treated with TQ for 24 hours was subjected to EMSA using probes derived from *DNMT1* promoter covering Sp1/NFkB binding elements. Note: lane 1, free probe; lanes 2 and 5, control; lanes 3 and 6, TQ (3 μM); lanes 3 and 7, TQ (10 μM). (**D**) ChIP assays using anti-NFkB (p65) and anti-Sp1 in MV4-11 cells treated with indicated amounts of TQ for 24 hours. The respective input DNA was used for normalization. (**E**) Western blot in MV4-11 and Kasumi-1 cells treated with TQ for 24 hours. The data represent three independent experiments; Data are mean ± SD; *p < 0.05.

As *DNMT1* gene abundance is an important regulator of DNA methylation [1, 2, 8, 24], and given that TQ impairs NFkB signaling, a *DNMT1* gene transactivator [1, 2, 24], we hypothesized that TQ treatment inhibits DNMT1 expression coupled with DNA hypomethylation. To test this, we treated leukemia cells, including ML-1, Kasumi-1 and MV4-11, with indicated doses of TQ and initially employed Western blot to assess DNMT1 changes. As expected, the expression of DNMT1, DNMT3a and Sp1, but not HSP90, was significantly decreased in these cell lines (Figure 2A), which was further verified in THP-1 and K562 cells upon exposure to TQ (not shown). As *miR-29b* directly binds to the 3′-UTR of *Sp1*, *DNMT3a* and *DNMT3b* [27, 28] thereby disrupting their expression, we measured *miR-29b* levels and observed an upregulation of *miR-29b* in the presence vs. absence of TQ (Figure 2B).

To dissect the molecular mechanisms underlying TQ-mediated *DNMT1* downregulation, we initially employed electrophoretic mobility shift assays (EMSA) [24, 27–29] and used the probes spanning the regions containing Sp1/NFkB binding elements in *DNMT1* promoter. As shown in Figure 2C, when the nuclear extracts (NE) prepared from MV4-11 and THP-1 cells were incubated with ^32^P-labled probes, Sp1/NFkB-DNA complex was detected in control group, but was significantly reduced in TQ-treated cells. This result supports the notion that Sp1/NFkB complex in D*NMT1* promoter was disrupted by TQ treatment, which might result from TQ-mediated Sp1 downregulation (see Figure 2A). To further verify the Sp1/NFkB complex in *DNMT1* promoter, we performed Chromatin Immunoprecipitation (ChIP) assays [3, 27, 30]. Briefly, the treated cells were fixed in formaldehyde, sonicated, immunoprecipitated by antibodies against Sp1 or the p65 subunit of NFkB. The precipitated DNA was qPCR-amplified using primers covering the Sp1/NFkB binding sites, and the results were normalized by the respective input DNA. TQ treatment abrogated the binding of Sp1/NFkB to *DNMT1* promoter in a dose-dependent manner, suggesting that TQ impairs *DNMT1* expression through Sp1 and NFkB dysfunction (Figure 2D). Because our studies and others demonstrated that Sp1/NFkB is also involved in the regulation of tyrosine kinase signaling [27, 31], as a proof of concept, we examined the changes of KIT and FLT3, the key regulators of leukemia pathogenesis. We found that exposure of Kasumi-1 and MV4-11 cells to TQ led to downregulation of KIT, FLT3, STAT5 and AKT followed by dephosphorylation of STAT5 and AKT (Figure 2E), indicating tyrosine kinase signaling as an additional molecular mechanism behind TQ-induced leukemia growth arrest, which merits a systematic characterization.

### TQ induces global DNA hypomethylation, blocks colony formation and promotes cell apoptosis *in vitro*

**Figure 3:**
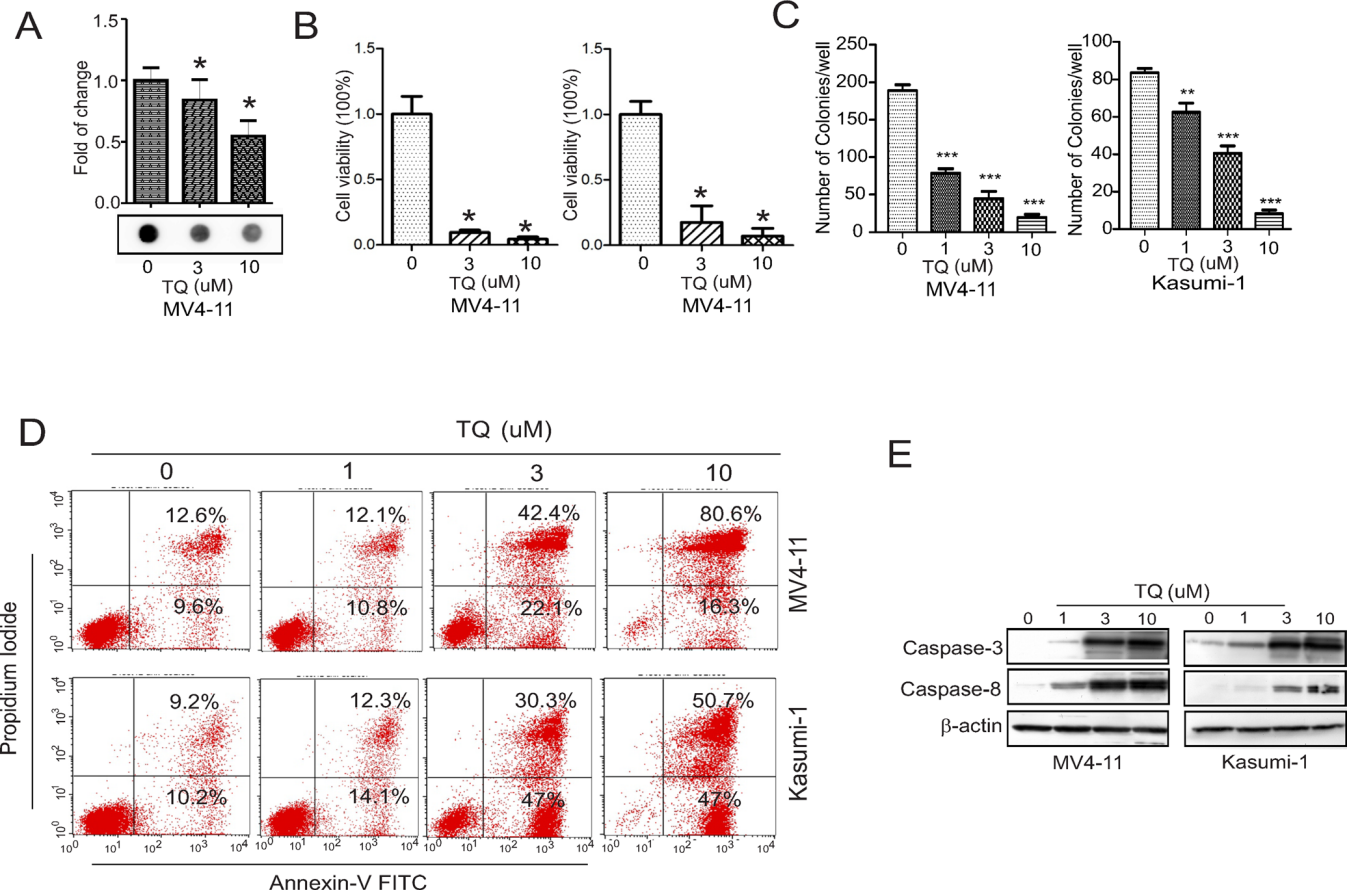
TQ inhibits DNA methylation and blocks leukemia growth *in vitro* (**A**) Dotblot assays using anti-5mC in MV4-11 cells treated with TQ for 48 hours. Upper, graph is the quantification of dot intensities; lower, the representative image of dotblot. (**B**) Kasumi-1 and MV4-11 cells were treated with TQ for 48 hours and the cell viability was determined by CCK-8 assays. (**C**) Kasumi-1 and MV4-11 cells were treated with TQ for 6 hours and subjected to colony formation assays. (**D**) Flow cytometry assays for cell apoptosis in Kasumi-1 and MV4-11 cells treated with TQ for 48 hours. (**E**) Western blot in Kasumi-1 and MV4-11 cells treated with TQ for 24 hours. The data represent three independent experiments; In CCK-8 assays, the experiments were done twice independently with 8 replicates in total; Data are mean ± SD; *p < 0.05, **p < 0.01, ***p < 0.001.

Considering that *DNMT1* gene abundance positively modulates DNA methylation levels [1, 2, 24], we next sought to address whether DNA methylation is decreased in the presence of TQ. In fact, the dotblot assays using anti-5mC revealed a significant reduction of DNA methylation in MV4-11 cells treated with TQ for 48 hours (Figure 3A). Because our previous studies suggested that DNMT1 upregulation [1] and DNA hypermethylation [1, 3, 8, 28] are significantly and positively associated with more aggressive leukemia growth and worse prognosis, we examined the growth status of TQ-treated cells. The results from CCK-8 assays disclosed a significant inhibition of cell proliferation (Figure 3B), and the colony assays identified a dose-dependent decrease of colony number (Figure 3C), when exposed to TQ. Further, flow cytometry assays found an enhanced cell apoptosis (Figure 3D), which possibly occurred via caspase activation, because Western blot showed that the active forms of caspase-3 and caspase-8 were significantly increased in the presence vs. absence of TQ (Figure 3E).

### TQ suppresses DNMT1-dependent DNA methylation and promotes cell apoptosis in leukemia blasts

**Figure 4:**
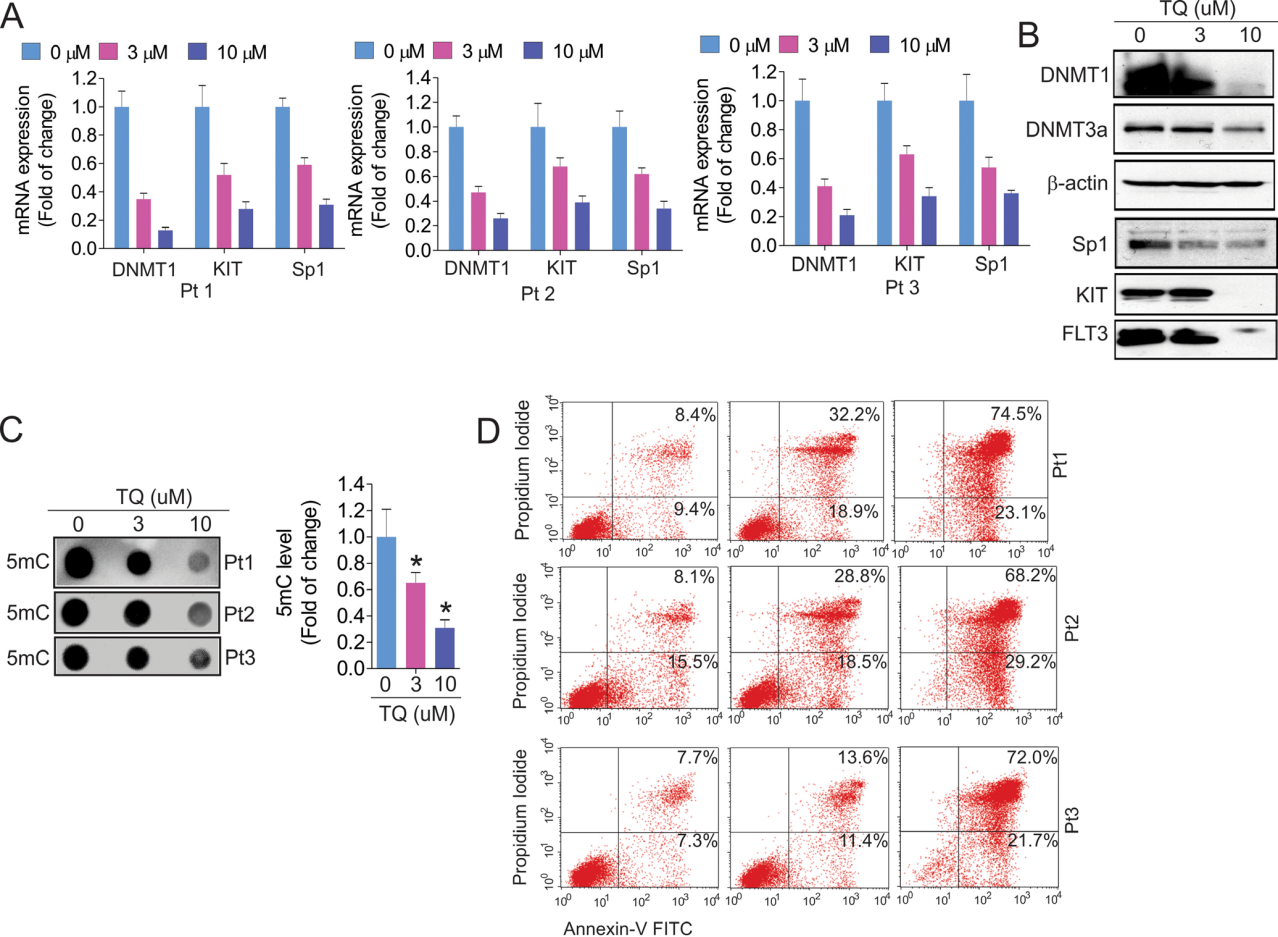
Exposure of leukemia primary cells to TQ leads to DNA demethylation and cell apoptosis The primary cells from leukemia patients (*n* = 3) were exposed to TQ for 24 hours. (**A**) qPCR showing the expression of TQ target genes. (**B**) The treated cells from three patients were pooled together and subjected to Western blot. (**C**) Dotblot using anti-5 mC measuring the change of DNA methylation. Graph is the quantification of dot intensities. Data are mean ± SD; *p < 0.05. (**D**) Flow cytometry assays for cell apoptosis in primary cells. Note: Pt, patient.

To explore the clinical implication of TQ as a DNA hypomethylating agent, we treated leukemia patient blasts with TQ. In agreement with the results from cell lines, TQ exposure significantly decreased the expression of DNMT1, DNMT3a, Sp1, KIT and FLT3 at both RNA (Figure 4A) and protein (Figure 4B) levels. This was followed by a significant decrease of global DNA methylation (Figure 4C) and an increase of cell apoptosis (Figure 4D).

### Characterization of TQ pharmacokinetics

To determine the pharmacokinetics of TQ in pre-clinical investigation, we have developed a LC-MS/MS method to quantify TQ in mouse plasma. As TQ has limited aqueous solubility for the *in vivo* study, we initially designed an appropriate formulation to dissolve TQ. When TQ was dissolved in ethanol/PEG400/saline (18/42/40 %), a clear solution of TQ in a concentration of 7.5–13 mg/ml was achieved, which was suitable for administration of 30–50 mg/kg dose in mice. Further, as TQ was light sensitive (not shown), we used the non-conventional method to extract TQ. Briefly, TQ was dissolved in Acetonitrile (ACN) to make stock solutions at 1 mg/mL. To test the linearity of the standard curve, 10 uL of the appropriate ranged (2–50 ng/mL) intermediated stock solutions was spiked into 100 uL of mobile phase, which was 50% ACN and 0.1% formic acid. The mixture was vortexed for 30 second and 20 μL was injected into the LC-MS/MS system. An Applied Biosystems Sciex API 3000 mass spectrometer (Applied Biosystems Sciex) equipped with an electrospray ionization (ESI) source was used for mass analysis and detection. The detector was operated at unit resolution in MRM mode using the transitions from the protonated molecular ions to product ions at m/z 164.00/134.10 for TQ. A SIL-10ADvp Shimadzu HPLC system (Shimadzu) consisting of system controller, degasser, binary pump and auto-sampler was used for solvent and sample delivery. The chromatographic separation was performed using a Beta Basic C8 column (2.1 mm × 50 mm, 5 μm) coupled with a Beta Basic C8 guard column (2.1 mm × 10 mm, 5 μm).

**Figure 5:**
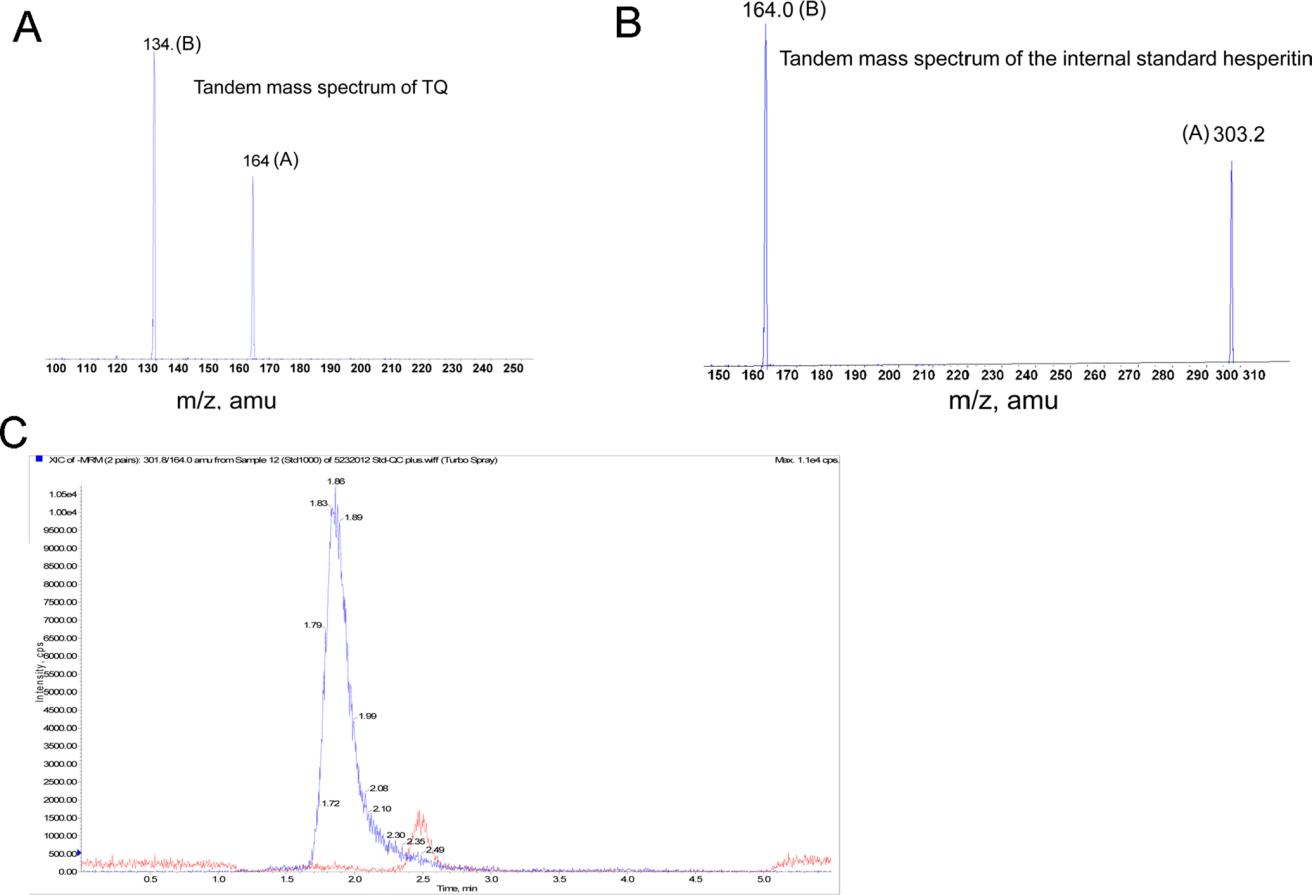
Sensitivity of the method at lower range standard curve (**A**) The protonated molecular ion (A) of TQ at m/z 164.0 and the collision assisted dissociation daughter ion (B, m/z 134.1) of the protonated molecular ion. (**B**) The protonated molecular ion (B) of the Internal Standard Hesperitin at m/z 303.2 and the collision assisted dissociation daughter ion (B) of protonated molecular ion at m/z 164.0. (**C**) The LC-MS/MS chromatogram of TQ and its I.S (Internal Standard) in mouse plasma. The retention time of TQ in mouse plasma is at about 2.5 min, and that of the internal standard is about 1.99 min.

**Figure 6:**
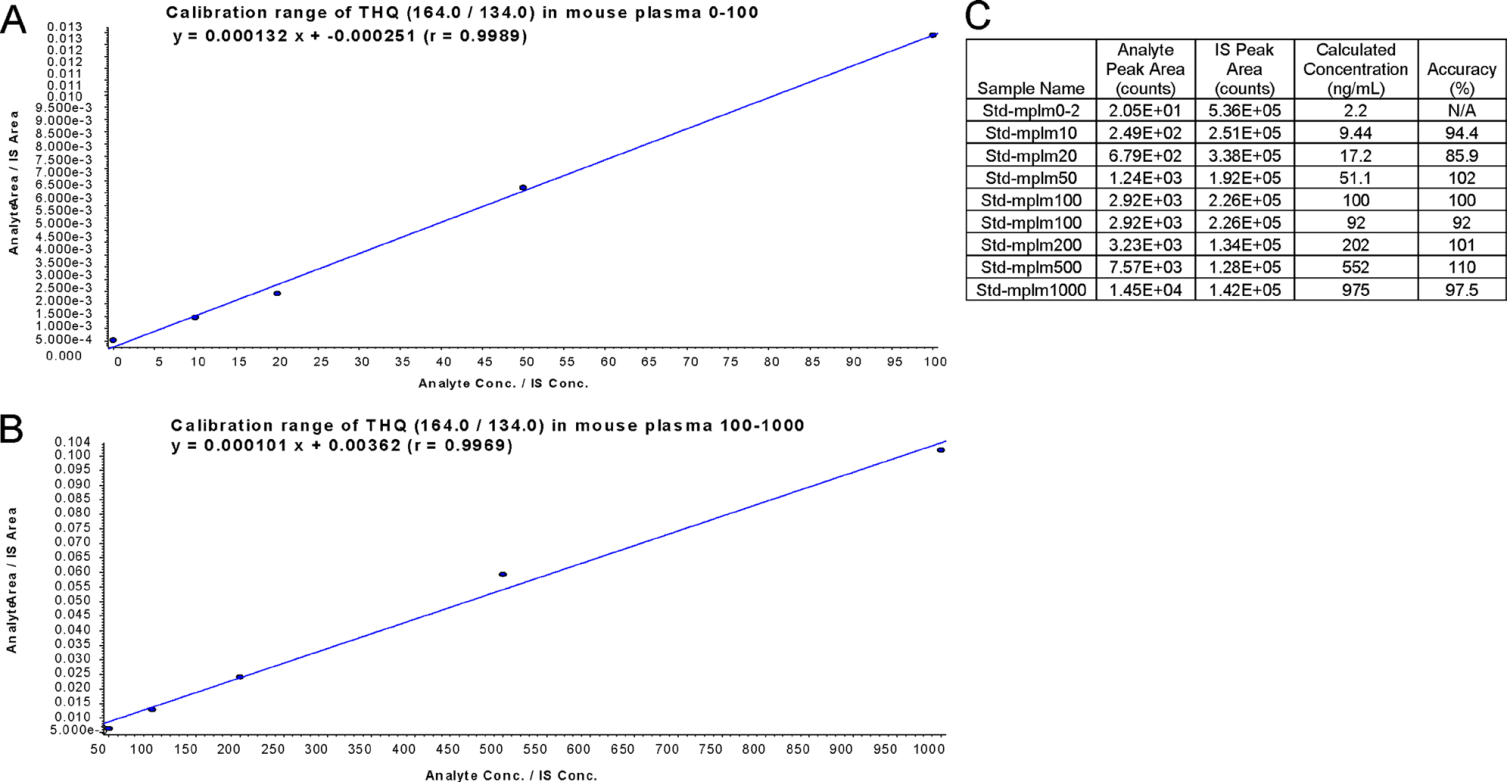
Calibration curve of TQ in mouse plasma (**A** and **B**) Calibration range of TQ (THQ) at 0–100 ng (**A**) and 50–1000 ng (**B**) in mouse plasma. (**C**) Calculated value and accuracy of calibration curve.

The mobile phases consisted of acetonitrile and 0.2% formic acid (FA) pumped at a flow rate of 0.2mL/min. The tandem mass spectrometry data of TQ and the assay at the low range calibration curve were shown in Figure 5A and 5B, respectively. The chromatogram of TQ with the internal standard in mouse plasma was shown in Figure 5C. Further, the linear range of calibration curve was from 0 to 1000 (ng/mL) in mobile phase solution and/or mouse plasma. The lowest concentration of detection (LOD) of TQ in mouse plasma was 5 ng/mL and the lowest concentration of quantification (LOQ) was 10 ng/mL. The calibration curve of TQ and the corresponding recovered concentration and accuracy in mouse plasma were shown in Figure 6A–6C. These data showed a linear range of the assay between 0–1000 ng/mL. The validation data showed that the percent of coefficient of variation CV and the accuracy values were 4.68%–9.19% and 95.5%–113% for the quality control (QC) concentrations of 10, 100, and 1000 ng/mL (*n* = 6), respectively. In addition, we also performed the stability study of TQ in mouse plasma. The stability data revealed that the compound was stable at −20°C under dim light condition, but not at 25°C and 37°C. Thus, TQ is more stable in the dark and at cold temperature.

### Plasma pharmacokinetic profile of TQ after I.V. injection

**Figure 7:**
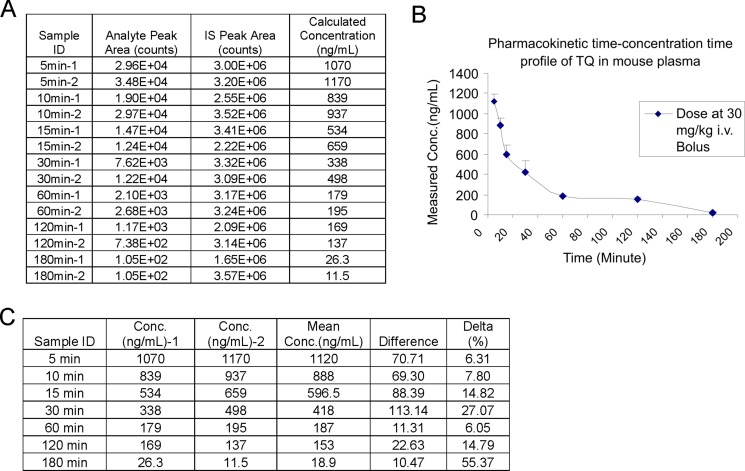
Plasma pharmacokinetic profile of TQ (**A**) The measured concentrations of TQ in CDF1 mouse plasma. (**B**) Pharmacokinetic concentration-time profiles of TQ in CDF1 mice (*n* = 2 mice/group) at 30 mg/kg. Data are mean ± SD of each time point. (**C**) The calculated concentrations of TQ in CDF1 mouse plasma. TQ was administered into the mice at the dose of 30 mg/kg/mouse. The mice were sacrificed at 5, 10, 15, 30, 60, 120 and 180 min post-injection.

To determine the plasma pharmacokinetics of TQ, we administered TQ, which was dissolved in a mixture of ethanol, PEG400 and saline at the concentration of 9.0 mg/mL, to the CDF1 male mice (*n* = 2 mice per time point) via the tail vein at the dose of 30 mg/kg/mouse. Plasma samples were collected at the time points of 5, 10, 15, 30, 60, 120 and 180 min post i.v. injection and stored under −80°C. These plasma samples were mixed with Hesperitin followed by the addition of ethyl acetate. After centrifugation at 14000 rpm, the supernatant was dried by N_2_, and then reconstituted in mobile phase solution. The concentration of TQ in each plasma sample was assessed by LC/MS. As shown in Figure 7A–7C, we successfully detected a dynamic change of TQ in mouse plasma. It seems that the plasma concentration-time profile follows a multi-exponential decline, likely to be triphasic.

### TQ administration attenuates leukemia growth in mice

**Figure 8:**
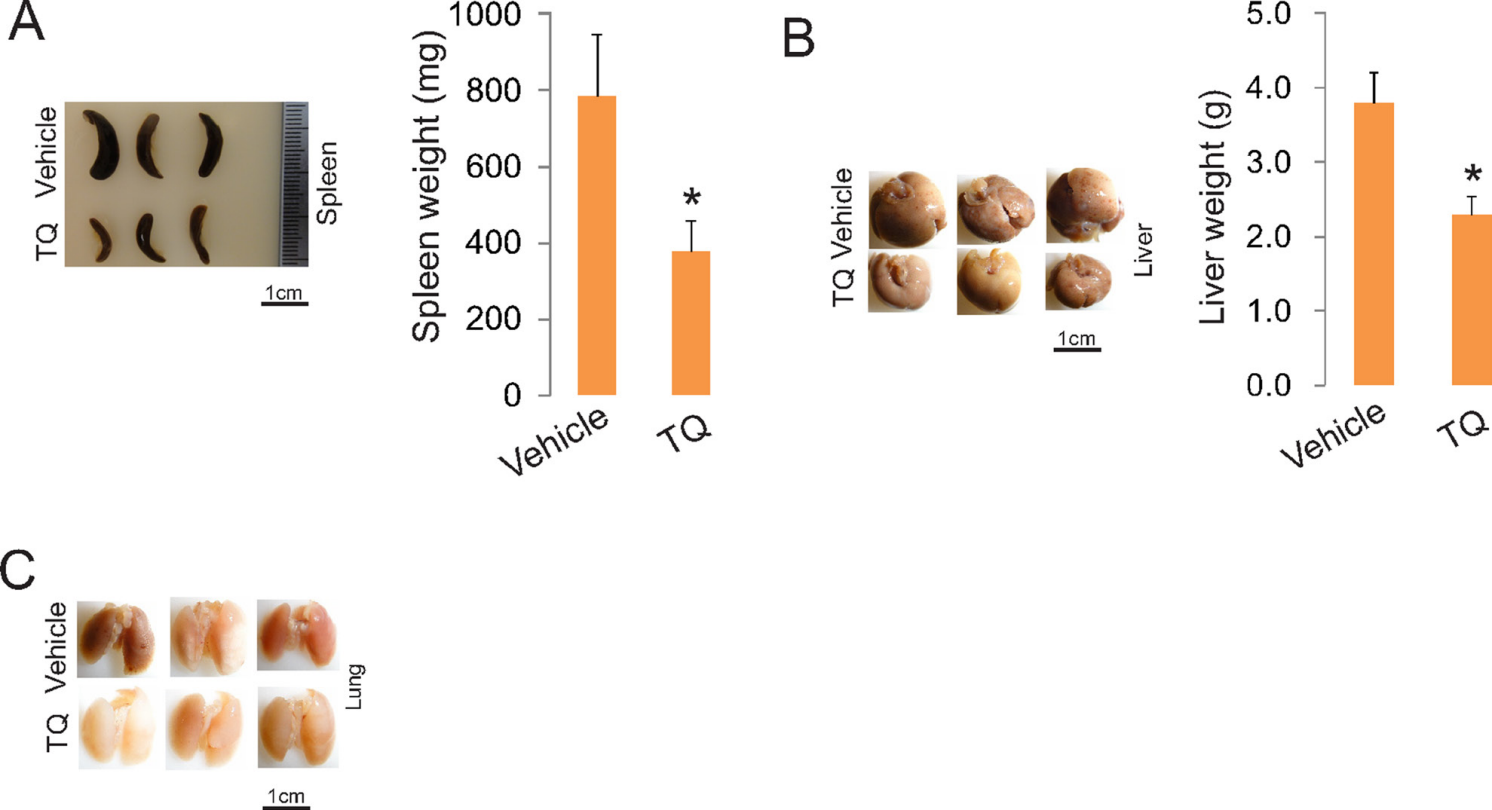
TQ administration inhibits leukemia growth *in vivo* C1498 cells (0.1 × 10^6^) were intravenously injected into C57BL/6 mice (*n* = 3 mice/group). One dose of 5 mg/kg TQ was given to these mice 12 hour prior to leukemia cell injection, which was followed by 2 times of 5 mg/kg/3-day in the first week and 2 times of 10 mg/kg/3-day in the second week. The experiments were terminated in three weeks and the organs were harvested. Left, external view of spleens, livers and lungs; right, graphs are the quantification of organ weight (*p < 0.05).

To demonstrate the anti-leukemia activities of TQ *in vivo*, we first determined a tolerable dose in mouse model for *in vivo* administration. In these experiments, we administered TQ into C57BL/6 mice (*n* = 10, 4–6 weeks old) by intravenous injection at the doses of 0, 15 and 30 mg/kg at normal pressure. Mice treated with equal volume of PBS + ethanol were used as controls. The schedule was 2 doses per week and completed 6 doses in 3 weeks. We observed that 100% mortality in mice receiving 30 mg/kg TQ, 30% mortality receiving 15 mg/kg and no mortality in control mice. Based on these results, we gave C57BL/6 mice (*n* = 3) one dose of 5 mg/kg TQ in PBS + ethanol 12 hour prior to i.v. injection of C1498 cells (0.1 × 10^6^) followed by 2 times of 5 mg/kg/3-day in the first week and 2 times of 10 mg/kg/3-day in the second week. The development of leukemic disease was monitored by the white blood cell count (not shown). We found that TQ administration induced leukemia regression, as indicated by the reversed splenomegaly (Figure 8A) and the inhibited leukemia growth in lungs and livers (Figure 8B and 8C). Collectively, TQ treatment impairs leukemia growth *in vitro*, *ex vivo* and *in vivo* in a mouse model of leukemia.

## DISCUSSION

Thymoquinone (TQ) is the main lipid constituent of *Nigella sativa* (black cumin) and *Monarda fistulosa*, which are widely known and used herbal medicine. Many reports showed that TQ has potent antitumor activity in both *in vitro* and *in vivo* for multiple types of cancer [32], but few studies focused on its potential anti-leukemia actions. Importantly, the molecular mechanisms underlying the anti-cancer activity of TQ and the methods to determine TQ pharmacokinetics in mouse plasma remain largely unexplored. In the current study, we present compelling evidence that TQ displays strong anti-leukemia activity *in vitro*, in leukemia patient primary cells and in a mouse model of leukemia. We identified DNMT1-assoicated DNA methylation as a hitherto unknown molecular rule mediating the anti-leukemia actions of TQ. Additionally, we have developed a LC-MS/MS method, which was validated for linearity and reproducibility, and successfully detected a dynamic change of TQ in mouse plasma after i.v. administration. Our findings shed new light on the mechanisms of TQ anti-cancer actions, providing a sound rationale in clinical trials for using TQ as a novel DNA hypomethylating agent to enhance the therapeutic index of typical DNA methylation inhibitors. Our results also offer an efficient method and platform for characterization of TQ pharmacokinetics that is critical for future clinical research.

Due to the significantly lower cytotoxicity to normal cells [32], TQ could be a promising anti-cancer drug by itself or in combination with other agents. To extrapolate it as a targeted therapy drug, the key is to uncover genes/signaling pathways that are responsible for TQ-induced cancer cell killing. While literature search has identified multiple mechanisms [32, 33], in the present study we shifted our attention to DNA methylation machinery. This is because 1) aberrant DNA methylation is a well-documented hallmark of cancers [34–36], 2) the therapeutic effects from typical DNA hypomethylating agents are limited [8, 37], 3) our previous study revealed that phytochemical compounds, such as curcumin, inhibit DNA methylation [25], and TQ is a phytochemical compound, and 4) multiple investigations demonstrated that TQ executes anticancer actions largely through NFkB signaling [38–41], which functions as a upstream regulator for many genes, including DNMT1 [1, 2, 24]. Indeed, we present evidence that TQ binds to the DNMT1 catalytic pocket, and dose-dependently suppresses DNMT1 methylation activity. We demonstrated that TQ incubation leads to downregulation of DNMT1, which, mechanistically, takes place through the disruption of Sp1/NFkB complex in the *DNMT1* gene promoter, and decrease of DNA methylation. Collectively, our findings support the hypothesis that TQ induces DNA demethylation by both binding DNMT1 protein and suppressing DNMT1 gene expression, which is similar to the mechanism of curcumin [25, 42], but different from that of decitabine requiring incorporation into newly synthesized DNA without obvious changes of DNMT1 gene transcription. These discoveries are not only in agreement with the documented role of NFkB as a central mediator of TQ anti-cancer actions [38–41] and a direct/indirect regulator of DNA methylation [1, 2, 24, 28], but also support DNMT1-associated DNA methylation as a new pharmacological endpoint of leukemia cells in response to TQ exposure as well as a common molecular rule underlying phytochemical compound-mediated cell death. To deepen insight into TQ's DNA hypomethylating activities for clinical implications, it is important for future studies to systematically define genes that are crucial for cancer pathogenesis and epigenetically reactivated by TQ treatment in distinct cancer cell/animal models, and correlates the findings with the progression of cancerous lesions. In addition, the exact binding model of TQ to DNMT1 remains unknown. Further exploration of the mode of binding to DNMT1 catalytic domain using mass spectrometry, structural analog probes, and enzymatic kinetics are warranted.

Considering that Sp1/NFkB is also involved in the regulation of receptor tyrosine signaling [27], we examined the changes of two receptor tyrosine kinases, KIT and FLT3, which play a pivotal role in leukemia pathogenesis [27, 31, 43]. We show that TQ treatment significantly impairs the expression of KIT and FLT3, leading to the dephosphorylation of their downstream effectors, such as STAT5 and AKT, in cell lines and patient blasts. These findings not only agree with previous findings showing that tyrosine kinase signaling represents one of various molecular rules behind TQ-impaired cancer cell growth [44], but also add new members to the TQ-targeted kinase family. Although further demonstrations are necessary, inactivation of KIT and FLT3 signaling by TQ may occur via their gene downregulation resulting from TQ-disrupted Sp1/NFkB functions. This mechanism is different from that of classical KIT and FLT3 inhibitors that competitively binds their enzymatic centers. Thus, these discoveries motivate us to consider a combination trial between TQ and inhibitors for KIT and FLT3 signaling in ongoing studies for improving therapeutic outcomes in leukemia.

## MATERIALS AND METHODS

### Cell lines and chemicals

Cell lines, Kasumi-1, MV4-11, THP-1 and ML-1, were purchased from The American Type Culture Collection (Manassas, VA), and maintained in RPMI 1640 medium, supplemented with 50 μg/mL streptomycin, 50 IU/mL penicillin plus 20% (Kasumi-1) or 10% (others) fetal bovine serum (FBS) at 37°C in 95% humidified atmosphere with 5% CO_2_ in air. Decitabine and thymoquinone (Sigma-Aldrich, MO) were dissolved in PBS or DMSO, respectively, as a stock solution, sterilized by filtration through a 0.22 μm syringe filter and stored at −80°C.

### Human DNMT1 homology modeling

The homology model of human DNMT1 catalytic domain was built with the crystal structure of bacterial modification methylase (Hhal) catalytic domain (PDB ID: 4MHT) as the modeling template. The 478 amino acids (AA 1139–1616) of DNMT1 were aligned against 327 residue Hhal methylase catalytic domain by a combination of CLUSTALW and Smith-waterman methods. The N- and C-terminal sequences align well with the template and the middle sequence has little homology between the two proteins. The two aligned regions were modeled using MODELLER v8 [45]. The resulting structure was minimized together with cofactor SAH and flipped cytosine substrate plus 15Å truncated octahedron TIP3P water box by using AMBER9 [46].

### Docking of thymoquinone

AutoDock version 4.0 [47] was used for the docking simulation. We selected the Lamarckian genetic algorithm (LGA) for ligand conformational searching because it has enhanced performance relative to simulated annealing or the simple genetic algorithm. For TQ, all hydrogens were added and Gasteiger charges were assigned, then non-polar hydrogens were merged. 80×100×70 3-D affinity grids centered on the empty binding site with 0.375 Å spacing were calculated for each of the following atom types: a) protein: A (aromatic C), C, HD, N, NA, OA, SA; b) ligand: C, A, OA, e (electrostatic) and d (desolvation) using Autogrid. The translation, rotation and internal torsions of the ligand are defined as its state variables and each gene represents a state variable. LGA adds local minimization to the genetic algorithm, enabling modification of the gene population. The docking parameters were as follows: trials of 100 dockings, population size of 250, random starting position and conformation, translation step ranges of 2.0 Å, rotation step ranges of 50°, elitism of 1, mutation rate of 0.02, crossover rate of 0.8, local search rate of 0.06, and 1 million energy evaluations. Final docked conformations were clustered using a tolerance of 1.5 Å root-mean-square deviations (RMSD).

### *In vitro* enzymatic activity assays

The DNMT1 enzymatic assays were performed as previously reported [25]. Briefly, a double strand DNA-oligonucleotide containing CCGG was labeled with 3′-biotin in one strand and 3′-digoxigenin-NHS ester in its complementary strand. This labeled probe was used as a substrate for M. SssI and the enzymatic assay was carried out in a solution containing SAM and the endonuclease HpaII. After cleavage by a HpaII, the 3′-digoxigenin-NHS ester was removed and no detectable fluorescence signal was detected, when anti-digoxigenin-AP antibody and the substrate Attophos were added to the solution. In contrast, when the CCGG sequence was methylated to CC^m^GG, cleavage activity of HpaII was inhibited and the 3′-digoxigenin-NHS ester was maintained leading to the generation of fluorescence signal. The methylation level of DNA probe was positively correlated with the intensity of the assay fluorescence signal, thereby the enzymatic activity of M. SssI.

### Quantification of DNA methylation

Genomic DNA was prepared using a DNeasy Tissue Kit (QIAGEN, Maryland). About 2 μg DNA was subjected to Dotblot using 5mC antibody as previously described [1–3, 48].

### Colony formation and flow cytometry assays

Methylcellulose colony formation assays were carried out in MethoCult^®^ mixture (Stem Cell Technologies) as previously described [1–3, 48]. Colonies were scored 7–14 days. Trypan blue exclusion was used to assess cell viability. Cell apoptosis assays were performed using Annexin V-PE Apoptosis Detection Kit I (BD PharmingenTM, San Diego, CA) according to the manufacturer's instruction, and followed by flow cytometry analysis.

### Cell counting Kit-8 (CCK-8) assays

After various treatments, the viable Kasumi-1 and MV4-11 cells were counted by the CCK-8 assay Kits (Dojindo Laboratories, Kumamoto, Japan) following the manufacturers’ instruction. Four wells were sampled per each experimental group in each experiment. Averages were reported as ± Standard Deviation (SD).

### Western blot

After various treatment, total protein lysates were prepared in 1× lysis buffer [20 mM HEPES (pH 7.0), 150 mM NaCl and 0.1% NP40] supplemented with 1 mM PMSF (Sigma Aldrich), 1× Phosphatase Inhibitor Cocktail 2 and 3 (Sigma Aldrich), and 1× protease inhibitors (protease inhibitor cocktail set III, Calbiochem-Novabiochem, San Diego, CA) and subjected to Western blot as previously described [1–3]. Equivalent gel loading was confirmed by incubating with β-actin antibody. The antibodies used are DNMT3a, Sp1 and β-actin (Santa Cruz Biotechnology, CA); DNMT1 and DNMT3b (Abcam, Cambridge, MA); KIT, FLT3, p-STAT5, STAT5, p-AKT, AKT, caspase-3 and caspase-8 (Cell Signaling Technology, Danvers, MA).

### Electrophoretic mobility-shift assays (EMSA)

Complementary oligonucleotides were synthesized (Sigma), annealed and labeled with ^32^P-dCTP and Klenow. Nuclear extracts were prepared from MV4-11 cells using Nuclear Extract Kit (Active Motif) in the presence of proteinase inhibitor cocktail (Roche Diagnostics, IN). EMSA with nuclear extracts and ^32^P-labeled probes were performed as described [24, 27–29, 49]. The oligo sequences used are: *DNMT1*/Sp1/1F: 5′-GGGCTCCGCGTGGGGGGGGTGTGTGCC CGCCTTGCGC-3′; *DNMT1*/Sp1/1R: 5′-GCGCAAGGCG GGCACACACCCCCCCCACGCGGAG-3′.

### Chromatin immunoprecipitation (ChIP)

ChIP assays were performed as described previously [3, 27, 30] using EZ-ChIP Assay Kit (Millipore, Billerica, MA). Briefly, about 2 × 10^6^ cells were cross-linked with 1% formaldehyde (Sigma-Aldrich), resuspended in 1% SDS lysis buffer and sonicated. After being precleared with protein G agarose, the lysates were immunoprecipitated by 5 μg antibodies for NFkB (Abcam) or anti-Sp1 (Santa Cruz Biotechnology). ChIP DNA was quantified by qPCR with SYBR^®^ Green PCR Master Mix. Fold change in binding was normalized by the respective input DNA. The primers are: DNMTCHF 5′-GTATCG CCTCTCTCCGTT-3′; DNMTCHR 5′-TCGGAGGCT TCAGCAGAC-3′.

### RNA isolation and quantitative PCR (qPCR)

According to the manufacturer's instructions, total RNA was isolated using miRNeasy Mini Kit (Qiagen, Valencia, CA) and cDNA synthesis was carried out using SuperScript^®^ III First-Strand Synthesis System (Invitrogen). The gene expression was assessed by SYBR^®^ Green qPCR and normalized by *18S* levels. For miRNA expression, qRT-PCR was performed by TaqMan MicroRNA Assays (Applied Biosystems, CA) and normalized by *U44/48* levels.

### Development of LC-MS/MS

### Sample preparation

To ensure stabilized condition for TQ during sample preparation, all samples were prepared under minimized light condition and in ice. Specifically, TQ was dissolved in acetonitrile (ACN) to make stock solutions at 1 mg/mL. The standard curve solutions of this analyte were prepared by serial dilution from the stock solutions in 50% acetonitrile to obtain the concentrations ranging from 0 to 1000 ng/mL. To test the linearity of this standard curve, 10 μL of the appropriate concentration range (2–50ng/mL) of the intermediated stock solutions were spiked into 100 μL of mobile phase which consists of 50% ACN in 0.2% formic acid. The mixture was vortex-mixed for 30 seconds and a 20 μL aliquot was injected into the LC-MS/MS system. In order to prepare the calibration curve sample, 10 μL of various standard curve solutions at 10, 20, 50,100, 200, 500, 1000, 2000, 5000 and 10000 ng/mL in 50% ACN was spiked into 100 μL mouse plasma containing 1000 ng/mL, then to the mixture was added 1 mL ethyl acetate and the mixture was vigorously vortex-mixed for 30 second followed by centrifugation at 12000 rpm for 3 min. The supernatant was then transferred into a new glass tube and dried by a stream of N_2_. The residue was reconstituted with 100 μL mobile phase (50% ACN and 0.2% formic acid), and a 20 μL aliquot was injected into an API-3000 triple quadruple mass spectrometer.

### LC-MS/MS conditions

An Applied Biosystems Sciex API 3000 triple quadruple mass spectrometer (Applied Biosystems Sciex, Ontario) equipped with an electrospray ionization (ESI) source was used for mass analysis and detection. The detector was operated at unit resolution in MRM mode using the transitions from the protonated molecular ions to product ions at m/z 164.00/134.10 for TQ.

A SIL-10ADvp Shimadzu HPLC system (Shimadzu, Columbia, MD) consisting of system controller, degasser, binary pump and auto-sampler was used for solvent and sample delivery. The chromatographic separation was performed using a Beta Basic C8 column (2.1 mm × 50 mm, 5 μm) coupled with a Beta Basic C8 guard column (2.1 mm × 10 mm, 5 μm). The mobile phases consisted of acetonitrile and 0.2% formic acid (FA) pumped at a flow rate of 0.2 mL/min.

### Plasma PK study of TQ

### Mouse treatment, plasma sample preparation and LC/MS analysis

CDF1 male mice (4–6 weeks old) were purchased from Harlan (Indianapolis, IN). Upon receipt, mice were acclimated before the study. All animal experiments were carried out according to a protocol approved by the Institutional Animal Care and Use Committee at The Ohio State University. The TQ solution was freshly prepared at the concentration of 9.0 mg/mL in the formulation of a mixture of ethanol, PEG400 and saline and the formulated solution (~100 μL or less) was administered to the mice via the tail vein at the dose of 30 mg/kg for each. Mice were sacrificed at the time points of 5, 10, 15, 30, 60, 120 and 180 min post i.v. injection. Each time point had 2 mice. Plasma samples were collected immediately at the time points mentioned above and frozen in a −80°C freezer until further analysis. The calibration curve was constructed in the concentration range of 0, 5, 10, 20, 50, 100, 200, 500, and 1000 ng/mL with QC in duplicates and the plasma PK samples were prepared in the same way as the calibration curve samples. Briefly, 100 μL of mouse plasma sample was mixed with 10 μL of 10 μg/mL of Hesperitin; then 1 mL ethyl acetate was added, followed by vigorous vortex mixing and the solutions centrifuged at 14000 rpm. The supernatant was separated and dried under N_2_, and then reconstituted in mobile phase solution. The concentration of TQ in each plasma sample was measured by LC/MS. The concentration–time data were analyzed by Excel software.

### Determination of the maximum tolerated dose

C57BL/6 mice (male, 4 -6 weeks old) were purchased from Harlan (Indianapolis, IN). All animal experiments were carried out according to a protocol approved by the Institutional Animal Care and Use Committee at The Ohio State University. In these experiments, TQ was dissolved in ethanol, diluted in PBS and administered into mice by intravenous (i.e., through the tail vein) injection in a small volume (0.2 ml) at normal pressure. Mice treated with equal volume of PBS (+ethanol) were used as controls. 10 mice per group was used and the drug doses were 0, 15 and 30 mg/kg. The administration schedule was 2 doses per week and completed 6 doses in 3 weeks.

### Leukemogenesis in mice

C57BL/6 mice (female, 4–6 weeks old) were purchased from The Jackson Laboratory (Bar Harbor, ME). All animal studies were performed with approval from the University of Minnesota Institutional Laboratory Animal Care and Use Committee. The C1498 cells (0.1 × 10^6^), a murine AML cell line, were intravenously injected into the C57BL/6 mice (*n* = 3). One dose of 5 mg/kg TQ was given to C57BL/6 mice 12 hour prior to C1498 cell injection, followed by 2 times of 5 mg/kg/3-day in the first week and 2 times of 10 mg/kg/3-day in the second week. The development of leukemic disease was monitored by the white blood cell count. The experiments were terminated in three weeks. Following sacrifice, the spleens, livers and lungs were harvested and weighted.

### Leukemia patients

The current study was approved by the University Institutional Review Board and conducted in accordance with the Declaration of Helsinki. All patients signed informed consent to store and use their leukemia tissue for discovery studies as part of Hematologic Malignancy Tissue Bank. Peripheral blood mononuclear cells (PBMCs) were isolated from whole blood using Ficoll-Paque™ and stored at −90°C. Samples were later thawed and recovered in RPMI 1640 medium with 20% FBS [1, 3] for 6 hours prior to treatment.

### Statistical analysis

The quantification for target changes was performed using the Student's t test. All statistical analyses were done using GraphPad Prism 5.0. Differences were considered statistically significant at *P* < 0.05. All *P* values were determined by unpaired, two-tailed Student's *t*-test.
